# Hepatitis A virus (HAV) packaging size limit

**DOI:** 10.1186/1743-422X-6-204

**Published:** 2009-11-18

**Authors:** Krishnamurthy Konduru, Siham M Nakamura, Gerardo G Kaplan

**Affiliations:** 1 Laboratory of Hepatitis and Related Emerging Agents, Center for Biologics Evaluation and Research, Food and Drug Administration, 8800 Rockville Pike, Bethesda, Maryland 20892, USA

## Abstract

**Background:**

Hepatitis A virus (HAV), an atypical Picornaviridae that causes acute hepatitis in humans, grows poorly in cell culture and in general does not cause cytopathic effect. Foreign sequences have been inserted into different parts of the HAV genome. However, the packaging size limit of HAV has not been determined. The purpose of the present study is to investigate the maximum size of additional sequences that the HAV genome can tolerate without loosing infectivity.

**Results:**

*In vitro *T7 polymerase transcripts of HAV constructs containing a 456-nt fragment coding for a blasticidin (Bsd) resistance gene, a 1,098-nt fragment coding for the same gene fused to GFP (GFP-Bsd), or a 1,032-nt fragment containing a hygromycin (Hyg) resistance gene cloned into the 2A-2B junction of the HAV genome were transfected into fetal Rhesus monkey kidney (FRhK4) cells. After antibiotic selection, cells transfected with the HAV construct containing the resistance gene for Bsd but not the GFP-Bsd or Hyg survived and formed colonies. To determine whether this size limitation was due to the position of the insertion, a 606 bp fragment coding for the Encephalomyocarditis virus (EMCV) internal ribosome entry site (IRES) sequence was cloned into the 5' nontranslated (NTR) region of HAV. The resulting HAV-IRES retained the EMCV IRES insertion for 1-2 passages. HAV constructs containing both the EMCV IRES at the 5' NTR and the Bsd-resistance gene at the 2A-2B junction could not be rescued in the presence of Bsd but, in the absence of antibiotic, the rescued viruses contained deletions in both inserted sequences.

**Conclusion:**

HAV constructs containing insertions of approximately 500-600 nt but not 1,000 nt produced viable viruses, which indicated that the HAV particles can successfully package approximately 600 nt of additional sequences and maintain infectivity.

## Background

Hepatitis A virus (HAV), a member of the *Picornaviridae *family, causes acute hepatitis in humans. The 27-32 nm non-enveloped HAV icosahedral particles encapsidate a 7.5 kb single-stranded positive-sense RNA genome [1], which contains a long open reading frame (ORF) flanked by 5' and 3' end non-translated regions (NTR). The long 5' NTR of approximately 750 nucleotides (nt) has a complex structure and contains an internal ribosome entry site (IRES) required for viral translation. The 3' NTR is short and ends in a poly(A) tail [2]. The HAV long ORF encodes a polyprotein of approximately 250 kDa that undergoes co- and post-translational processing into smaller structural (VP0, VP3, and VP1-2A) and non-structural (2B, 2C, 3A, 3B, 3C, and 3D) proteins [3,4]. HAV 3C is a cysteine proteinase (3C^pro^) responsible for most of the polyprotein cleavages and is the only protease coded in the HAV genome [5-9]. The 2A-2B junction is the primary cleavage site of the HAV polyprotein processed by 3C^pro ^[9,10]. The VP0 undergoes structural cleavage, and an unknown host cellular protease cleaves the VP1-2A junction [11].

HAV is a hepatotropic virus transmitted through the fecal-oral route. Pathogenesis of HAV is poorly understood, and it is unclear whether the virus needs to replicate in extra-hepatic sites before reaching the liver. After binding to its cellular receptor HAVCR1 [12,13], the HAV genome is delivered to the cytoplasm by an unknown mechanism. Once in the cytoplasm, the HAV genome is translated, transcribed, and encapsidated without in general causing cytopathic effect. The virus is eliminated by the immune system and does not establish chronic infection. Inactivated HAV vaccines are safe and effective, and are currently used in most of the world to prevent and treat HAV infection [1,14,15].

Considerable interest has been devoted to develop HAV as an expression vector for combination vaccines, expression of proteins in the liver, and basic research on this poorly understood human pathogen. We have previously shown that replication-competent HAV constructs containing inserts of 60-81 nt coding for malaria and FLAG-tag epitopes at the N-terminus of the HAV polyprotein were stable for at least 6 passages [16]. An HAV recombinant containing 420-nt insertion at the 2A-2B junction was stable for up to five passages [10]. HAV constructs carrying a seven amino acid human immunodeficiency virus gp41 epitope at the surface of the HAV particles elicited an immune response against gp41 in infected animals [17,18]. Recently, we showed that a 456-nt fragment coding for a blasticidin (Bsd) resistant gene inserted at the 2A-2B junction of wild type HAV was stable for 9 passages [19], conferred Bsd resistance to infected cells, and was used to develop an antibiotic resistance titration assay to evaluate anti-HAV antibodies in preparations of human immunoglobulins [20]. Although foreign sequences have been successfully inserted into different parts of the HAV, the packaging size limit of HAV has not been determined. To study the maximum size of foreign sequences that HAV could tolerate, we cloned exogenous sequences into the 2A-2B junction and/or N-terminus of the polyprotein. A 456-nt fragment coding for a Bsd resistance gene was engineered into the 2A-2B junction of HAV and maintained for 25 passages with or without antibiotic selection. A recombinant HAV containing the Encephalomyocarditis (EMCV) internal ribosome entry site (IRES) of 606 nt cloned at the 5'NTR between the HAV IRES and the initiation codon of the HAV polyprotein only maintained the insert for a few passages. However, recombinant HAV constucts containing approximately 1,000 nt at the 2A-2B junction or both the EMCV IRES at the 5'NTR and the Bsd resistance gene at the 2A-2B junction could not be rescued from transfected cells. Our results indicated that HAV has a restricted packaging size limit that can accommodate approximately 600 nt of additional sequences.

## Results

### Insertions at the 2A-2B junction

**Figure 1:**
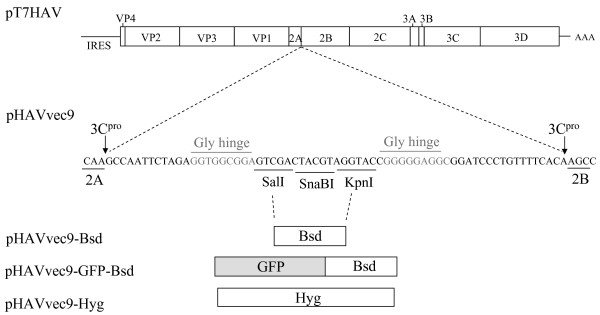
**Schematic representation of HAV constructs containing insertions in the 2A-2B junction**. Antibiotic resistance genes were cloned the 2A-2B junction of the HAV genome in pHAVvec9 [19]. This plasmid contains a polylinker coding for *SalI, SnaBI*, and *KpnI *restriction sites flanked by Gly hinges (grey color letters) and HAV 3C^pro ^cleavage site (arrows) engineered into 2A-2B junction of pT7HAV. The blasticidin resistance gene (Bsd) coding for blasticidin deaminase, green fluorescent protein (GFP)-Bsd fusion protein, and hygromycin resistance gene (Hyg) coding for hygromycin phosphotransferase were cloned into the *SalI *and *KpnI *sites in the polylinker of pHAVvec9, and the resulting plasmids were termed pHAVvec9-Bsd, pHAVvec9-GFP-Bsd, and pHAVvec9-Hyg, respectively.

**Figure 2:**
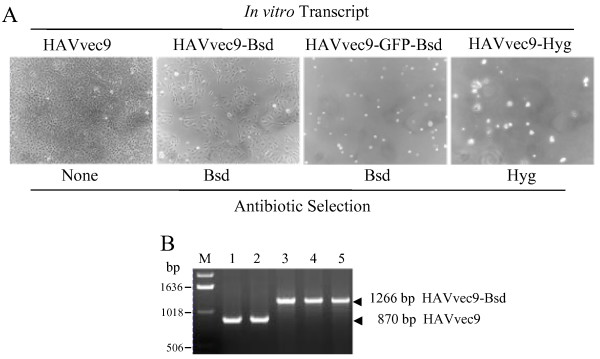
**Rescue and stability of the HAV constructs containing antibiotic resistance genes in the 2A-2B junction**. (**A**) Subconfluent FRhK4 cells were transfected with in vitro T7 polymerase transcripts of pHAVvec9, pHAVvec9-Bsd, or pHAVvec9-GFP-Bsd, or pHAVvec9-Hyg or mock-transfected. Cells were and incubated for 2-weeks in selection medium containing 1 μg/ml Bsd for all transfections except for cells transfected with pHAVvec9-Hyg transcripts, which were grown in the presence of 100 μg/ml Hyg. Phase contrast micrographs were taken with a Zeiss Axiovert microscope at 200× magnification. (**B**) Stability of the recombinant HAV. Viral RNA was extracted and fragments were amplified by RT-PCR using HAV primers forward VP1 coding for nts 2928-2951 and reverse 2B primer coding for nts 3715-3738. RT-PCR fragments amplified from RNA extracted from HAV/7 (lane 1), HAVvec9 (lane 2), HAVvec9-Bsd passage 1 (lane 3), and HAVvec9-Bsd passage 25-times in the presence (lane 4) or absence (lane 5) of Bsd were analyzed by TAE-1%-agarose gel electrophoresis. The RT-PCR fragments from HAVvec9-Bsd and HAVvec9 are indicated by arrowheads and their sizes given in base pairs (bp). The size of the DNA molecular weight markers (lane M) is indicated in bp.

To determine the packaging size limit of the HAV genome, we inserted foreign sequences into a 66-nt polylinker engineered between 3C^pro ^cleavage sites at the 2A-2B junction of the HAV cDNA in pHAVvec9 [19] (Figure 1). A 396-nt fragment containing a Bsd resistance gene coding for a Bsd deaminase, a 1098-nt fragment coding for the same resistance gene fused to the GFP protein (GFP-Bsd), or a 1032-nt fragment containing a hygromycin (Hyg) resistance gene coding for a hygromycin-phosphotransferase were inserted into the polylinker of pHAVvec9, and termed pHAVvec9-Bsd, pHAVvec9-GFP-Bsd, and pHAVvec9-Hyg, respectively (Figure 1). All the inserts lacked translation initiation and termination codons to allow expression of the coded proteins as part of the HAV polyprotein. To rescue viruses, T7 polymerase *in vitro *transcripts of the HAV constructs were transfected into FRhK4 cells. A day after transfection, cells were split 1:6 and grown in medium that contained Bsd 0.5-3 μg/ml or hygromycin 25-250 μg/ml. As expected, some cells transfected with transcripts from pHAVvec9-Bsd survived selection with 1 μg/ml Bsd and developed colonies (Figure 2A). However, cells transfected with the T7 *in vitro *transcripts from pHAVvec9-GFP-Bsd did not survive antibiotic selection, show GFP fluorescence, or produce infectious virus even without Bsd selection. Cells transfected with *in vitro *transcripts from pHAVvec9-Hyg also did not survive antibiotic selection. A virus stock termed HAVvec9-Bsd was prepared from these Bsd-resistant cells and used to infect naïve FRhK4 cells in the presence of 2 μg/ml Bsd. Cells infected with HAVvec9-Bsd but not mock-infected cells survived selection with Bsd, indicating that HAVvec9-Bsd was infectious. However, HAVvec9-Bsd produced 1-1.5 log less virus than HAVvec9 and parental HAV/7 [19]. The stability of Bsd gene in HAVvec9-Bsd virus was assessed during 25 serial passages in the absence of Bsd. RT-PCR (Figure 2B) and nucleotide sequence analysis (data not shown) revealed that the Bsd gene insert was stable during the 25 serial passages in FRhK4 cells. These data suggested that HAV could tolerated insertions of approximately 500-1,000 nt at the 2A-2B junction

### Insertions at the 5' NTR

**Figure 3:**
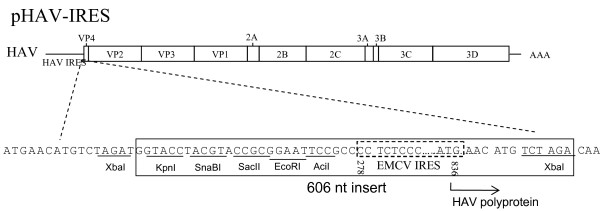
**Schematic representation of the HAV construct containing the EMCV IRES at the 5'NTR of the HAV genome**. A 606-nt cDNA fragment containing the EMCV IRES was inserted into the HAV 5'NTR between the HAV IRES and the initiation codon (ATG) of the HAV polyprotein. The resulting construct was termed pHAV-IRES, and contained a short synthetic polylinker containing *KpnI, SnaBI, SacII, EcoRI*, and *AciI *restriction sites followed by nts 278-836 of the EMCV IRES (dashed rectangle) inserted into the *XbaI *site in VP4 in such a way as to recreate the native initiation codon of the HAV polyprotein. The mature HAV proteins and the viral poly (A) tail (AAA) are indicated in the schematic representation of the HAV genome organization.

**Figure 4:**
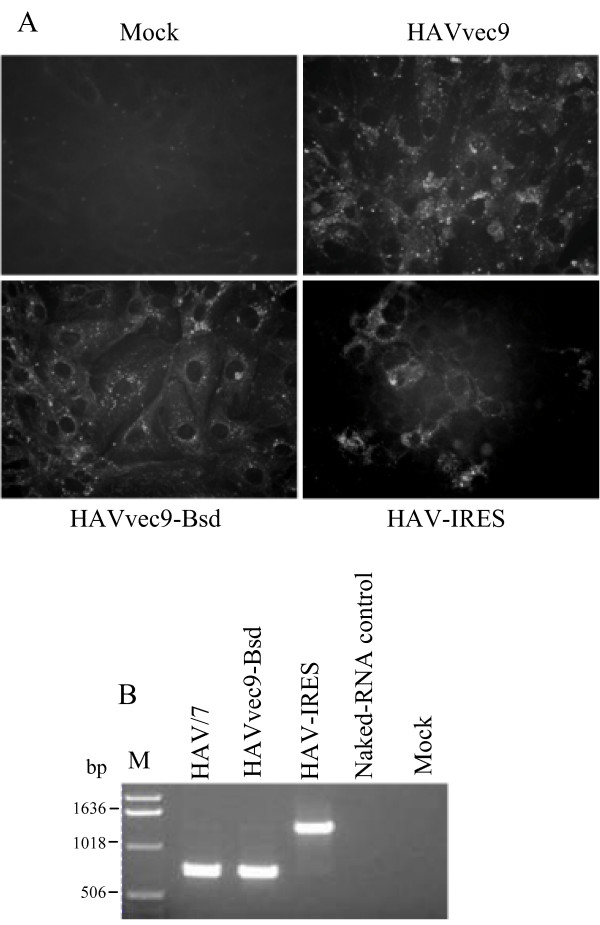
**Rescue and stability of the HAV constructs containing the EMCV IRES at the 5'NTR**. (**A**) IF analysis of FRhK4 cells transfected with *in vitro *RNA transcripts from pHAVvec9, pHAVvec9-Bsd, or pHAV-IRES or mock-transfected. Cells were fixed with acetone two weeks post-transfection, and stained with anti-HAV neutralizing monoclonal antibodies K2-4F2 and K3-4C8 and FITC-conjugated goat anti-mouse antibodies. Micrographs were taken with a Zeiss microscope at 400× magnification. (**B**) Analysis of the stability of HAV recombinants containing the EMCV IRES. RT-PCR analysis of genomic RNA extracted form HAV/7, HAV-IRES, HAVvec9-Bsd virions and amplified using primers corresponding to nts 484-507 and 1167-1194 of HAV. As negative control, T7 polymerase *in vitro *transcripts from pHAV-IRES were spiked into media, layered on top of a 40% sucrose cushion, and sedimented by ultracentrifugation. RNA extracted from the pellet was used for the RT-PCR analysis (Naked-RNA control). The size of the DNA molecular weight markers (lane M) is indicated in bp.

To assess whether the packaging size limitation of 500-1,000 nt was site-specific, we inserted a nt fragment into the 5' NTR of the HAV genome between the HAV IRES and the initiation codon of the HAV polyprotein. We chose the EMCV IRES because it is a strong ribosome entry site compared to the weak HAV IRES [21,22] and has been widely used in other viral systems [23-29]. We hypothesized that a virus with both IRESes would have a translation advantage compared to the HAV IRES alone. The EMCV IRES 606-nt fragment was cloned adjacent to the polyprotein initiation codon to assure that it would drive translation of the HAV polyprotein (Figure 3). FRhK4 cells were transfected with T7 transcripts of the double IRES HAV construct. After two weeks of incubation, IF analysis revealed that 10% of the cells had the characteristic cytoplasmic granular fluorescence of HAV-infected cells compared to 100% of the cells transfected with pHAVvec9 or HAVvec9-Bsd transcripts (Figure 4A). A virus stock was prepared and termed HAV-IRES, and the presence of the EMCV IRES was assessed by RT-PCR analysis (Figure 4B). To do so, viral particles were purified by sedimentation through a 40% sucrose cushion, and viral RNA was extracted from the pellet. As a negative control, the same amount of transfected T7 RNA transcripts (20 μl) was purified in parallel by sedimentation through a 40% sucrose cushion, and RNA was extracted from the pellet. As a positive RT-PCR control, we used RNA extracted from purified HAV/7 particles. RT-PCR analysis showed that the HAV-IRES but not HAV/7 particles contained the expected 600 nt EMCV IRES fragment, which was verified by automated nucleotide sequencing. RT-PCR fragments were not amplified from the negative control sample. These data indicated that the 606-nt EMCV IRES insert was packaged into the HAV-IRES particles. To further assess the stability of the inserted EMCV-IRES, we performed serial passages of HAV-IRES in FRhK4 cells at weekly intervals. At each cell passage, a virus stock was produced and analyzed by RT-PCR as described above. After 2 passages, most of the EMCV IRES was deleted leaving only a small insert of 21 nt (data not shown). These data indicated that HAV was capable of packaging the 606-nt insert coding for the EMCV IRES but that HAV-IRES was an unstable recombinant virus.

### Insertions at both the 2A-2B junction and the 5'NTR

**Figure 5:**
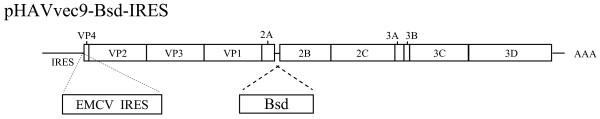
**Schematic representation of the HAV construct containing the EMCV IRES at the 5'NTR and the Bsd resistance gene at the 2A-2B junction**. The EMCV IRES was inserted into the HAV 5'NTR in pHAVvec9-Bsd. The construct containing both the EMCV IRES at the 5'NTR and the Bsd resistance gene at the 2A-2B junction was termed pHAVvec9-Bsd-IRES.

**Figure 6:**
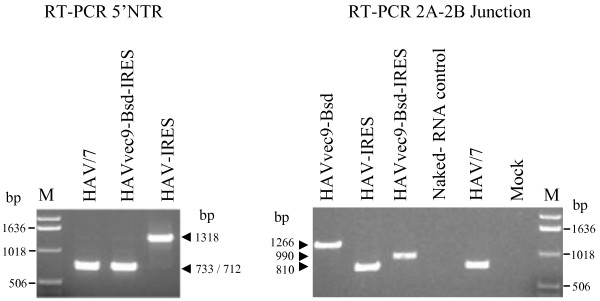
**Rescue and stability of the HAV constructs containing the EMCV IRES and Bsd resistance gene**. Viral RNA was extracted from purified virions and analyzed by RT-PCR as described in Figures 2B and 4B.

To rule out that constrains at the specific insertion sites unrelated to packaging limited the viability of the HAV constructs, we cloned both the Bsd resistance gene into the 2A-2B junction and the EMCV IRES into the 5' NTR (Figure 5). T7 polymerase *in vitro *transcripts of the resulting construct, pHAVvec9-Bsd-IRES, were transfected into FRhK4 cells. Transfected cells did not survive selection 1 μg/ml Bsd indicating that HAV did not tolerate the double insertion. IF analysis of transfected cells grown in the absence of Bsd selection showed that less than 10% of cells contained HAV antigens (data not shown). RT-PCR analysis of RNA extracted from particles purified by sedimentation through a 40% sucrose cushion revealed that the EMCV IRES and the Bsd resistance gene contained deletions (Figure 6). Nucleotide sequence analysis of PCR fragments showed the presence of only 21 nt from the EMCV IRES at the 5'NTR and 120 nt from the Bsd resistance gene at 2A-2B junction. Interestingly, the polylinker, 3C^pro ^cleavage sites, and Gly hinge flanking sequences were preserved. These data indicated that HAV cannot tolerate insertions larger than 600 nucleotides, and that this limitation in size is not due to site-specific restrictions of the inserted sequences.

## Discussion

In this paper we studied the packaging size limit of HAV. The virus tolerated the insertion of approximately 500 nt at the 2A-2B junction and 600 nt at the 5'NTR. The insertion of 456-nt fragment coding for a Bsd resistance gene flanked by 3C^pro ^cleavage sites was remarkably stable for 25 passages even in the absence of antibiotic. However, HAV could not accommodate larger inserts in the same site, and viruses containing GFP-Bsd and Hyg selectable markers could not be rescued from transfected cells. It should be pointed out FRhk4 cells transfected with eukaryotic expression vectors containing the GFP-Bsd and Hyg resistance genes survived selection with Bsd and Hyg, respectively. Moreover, FRhK4 cells transfected with an eukaryotic expression vector containing the GFP-Bsd fusion protein fluoresced similarly to cells transfected with a construct containing only GFP (data not shown). Since HAVvec9-Bsd was very stable, it is unclear why we could not rescue an HAV construct containing GFP-Bsd. One simple explanation is that we exceeded the packaging size limit of HAV. However, cells transfected with pHAVvec9-GFP-Bsd *in vitro *transcripts did not survive selection with Bsd suggesting that packaging alone was not the only factor affecting infectivity. Therefore, we hypothesized that site-specific or sequence-specific constraints at the 2A-2B junction site prevented HAV replication. To test this hypothesis, we introduced a completely different sequence at another site of the HAV genome. To that effect, we cloned a 606-nt fragment coding for the EMCV IRES between the HAV IRES and the initiation codon of the HAV polyprotein. Because HAV containing the EMCV IRES was unstable and tended to delete the insert in a few passages, we limited our study to viruses produced at the initial passage. The failure to rescue virus from the construct containing the Bsd resistance gene at the 2A-2B junction and the EMCV IRES at the 5'NTR indicated that the maximum size of foreign sequences that can be incorporated into the HAV genome of a viable virus is approximately 600 nt. Our study showed that HAV can package genomes of approximately 8,100 nt composed of the full-length genome and an insertion of approximately 600 nt, which can accommodate an additional 200 amino acid protein into the HAV polyprotein. Although the size of the extra sequences is somehow limited, HAV could be used as an expression vector for the development of combination vaccines and delivery of genes to the liver.

## Conclusion

In this study we showed that viable HAV can package full-length viral genomes containing insertions of approximately 600 nt.

## Methods

### Cells and viruses

Fetal rhesus monkey kidney (FRhK4) cells, a gift of S. Emerson, National Institutes of Health (NIH), were grown in Dulbecco's modified Eagle's medium supplemented with 10% fetal bovine serum. The cell culture-adapted human HM-175 strain of HAV was derived from an infectious cDNA clone [30], termed HAV/7 [31], and grown in FRhK-4 cells. Nucleotide positions of the HAV genome are according to the published HM175 HAV cDNA sequence [32].

### Plasmid constructions

Standard molecular biology methods [33] were used to construct the HAV recombinants. PCR-based DNA fragments were amplified using expand high fidelity PCR kit (Roche) in 25 cycles consisting of 95°C for 30 sec, 55°C for 1 min, and 72°C for 2-3 min. For overlap PCR, DNA fragments were denatured at 94°C and annealed at 45°C for 2 min in each step. The blasticidin (Bsd), fusion of green fluorescent protein with Bsd (GFP-Bsd), and hygromycin (Hyg) resistance genes were generated by PCR with eliminating the translation initiation (ATG) and termination codons and introducing 5' *SalI *and 3' *KpnI *restriction sites for cloning into the polylinker at the 2A-2B junction of the HAV polyprotein in pHAVvec9 [19]. To clone inserts in-frame at the 2A-2B junction, PCR products and pHAVvec9 were digested with *SalI *and *KpnI*, gel purified, and ligated. The oligonucleotides used to generate the PCR fragments are described in Table 1. The sequences inserted into the HAV geome are described in Table 2. Constructs were verified by automated nucleotide sequence analysis. The following plasmids were used in this work:

**Table 1:** PCR Primers

Name*	PCR Fragment	**Oligonucleotide Sequence**^&^
Bsd forward (SalI)	Bsd	5'-GTCGACGTCGACCAGGCCAAGCCTTTGTCTCAAGAA-3'

Bsd reverse (KpnI)	Bsd	5'-CGGTTAGGTACCGCCCTCCCACACATAACCAGAGGG-3'

GFP forward (SalI)	GFP-Bsd	5'-GTCGACGTCGACGCCTCCAAAGGAGAAGAACTTTTC-3'

Hyg forward (SalI)	Hyg	5'-GTCGACGTCGACAAAAAGCCTGAACTCACCGCGACG-3'

Hyg reverse (KpnI)	Hyg	5'-CGGTTAGGTACCGTTAGCCTCCCCCATCTCCCGATC-3'

EMVC IRES forward (XbaI)	EMCV IRES	5'-TTAGTCTAGATGGTACCTACGTACCGCGGAATTCCGCCCCTCTCCCTAACGTTACTGGCCGAA-3'

EMVC IRES reverse (XbaI)	EMCV IRES	5'-TTTCTAGACATGTTCATATTATCATCGTGTTTTTCAA-3'

*Restriction enzymes in brackets cut the PCR fragment amplified with the oligonucleotides.

^&^The restriction site in each oligonucleotide is underlined.

**Table 2:** Insertions in the HAV 2A-2B junction or 5'NTR

Construct	Insert	Site	Size (nt) *	**Infectivity**^&^
pHAVvec9	polylinker	2A-2B	66	+
pHAVvec9-Bsd	Bsd	2A-2B	396	+
pHAVvec9-GFP-Bsd	GFP-Bsd	2A-2B	1098	-
pHAVvec9-Hyg	Hygromycin	2A-2B	1032	-
pHAV-IRES	EMCV IRES	5'NTR	606	+
pHAVvec9-Bsd-IRES	Bsd	2A-2B	396	-
	EMCV IRES	5'NTR	606	

* pHAVvec9-Bsd, pHAVvec9-GFP-Bsd, and pHAVvec9-Hyg, and pHAVvec9-Bsd-IRES contain additional 60-nt corresponding to the polylinker inserted at the 2A-2B junction.

^&^Infectivity (+) or lack of infectivity (-) of the T7 polymerase *in vitro *synthesized transcripts from the plasmid constructs was assessed in FRhK4 cells by IF analysis.

pT7HAV contains the infectious cDNA cell culture-adapted HM-175 strain of HAV under the control of a T7 RNA polymerase promoter in pGEM1, and the *in vitro *transcripts were infectious in FRhK4 cells [16].

pHAVvec9 was derived from pT7HAV as described previously [19]. pHAVvec9 contains a polylinker with unique SalI, SnaBI, and KpnI restriction sites.

pHAVvec9-Bsd contains Bsd gene cloned into the polylinker of the HAV cDNA in pHAVvec9 [19].

pHAVvec9-GFP-Bsd contains a GFP-Bsd fusion cloned into the HAV cDNA polylinker in pHAVvec9. The GFP-Bsd fusion was amplified by PCR from pTracer-CMV/Bsd (Invitrogen). To create unique *SalI *site in GFP-Bsd insert, a silent substitution mutation (from GT**C**GAC to GT**A**GAC) was introduced to eliminate *SalI *site in the *GFP *gene sequence using overlap PCR.

pHAVvec9-Hyg contains a Hyg resistance gene cloned into the HAV cDNA polylinker in pHAVvec9. The Hyg resistance gene was amplified by PCR from pCFB-EGSH (Stratagene).

pHAV-IRES contains the EMCV IRES (corresponding to 278-836 nt of the EMCV genome) cloned between the HAV IRES and the HAV polyprotein initiation codons in pT7HAV (Figure 3). To construct pHAV-IRES, a 606-nt cDNA fragment containing nt 278-836 of EMCV flanked at the 5' end by a *XbaI *site and at the 3'end by the two tandem initiation codons of the HAV polyprotein and an *XbaI *restriction site was amplified from pCITE-1 (Novagen), a plasmid containing the EMCV IRES. This amplified cDNA fragment was cut with XbaI and cloned into the unique XbaI site adjacent to the HAV initiation codons in pT7HAV.

pHAVvec9-Bsd-IRES was constructed by cloning the EMCV IRES from pHAV-IRES into pHAVvec9-Bsd. To do so, the 1,783-nt *BspEI *and *BstEII *fragment of pHAV-IRES containing the EMCV IRES was cloned into pHAVvec9-Bsd cut with *BspEI *and *BstEII *at nts 24 and 1194 of the HAV cDNA, respectively. The resulting construct contained both the EMCV IRES and the 5'NTR and the Bsd resistance gene at the 2A-2B junction of the HAV cDNA.

### *In vitro *RNA synthesis and transfection

Synthesis of full-length HAV RNA transcripts was performed using T7 RNA polymerase [16]. To do so, plasmids were linearized with *HaeII*, which cuts immediately downstream of the poly(A) of the HAV cDNA [16,30]. Subconfluent FRhK4 cells in 25-cm^2 ^flasks were transfected with *in vitro *synthesized RNA transcripts using DEAE-dextran as facilitator [34] or Lipofectamine 2000 (Invitrogen). After 30 min incubation at room temperature, fresh media was added to replace transfection solution and incubated at 35°C. Cells were split weekly into new flasks, and an aliquot was passed to 8-well chamber slides for immunofluorescence (IF) analysis. To prepare viral stocks, monolayers with approximately 80% of the cells expressing HAV antigens as assessed by IF analysis were subjected to three freeze-thaw cycles and clarified by low-speed centrifugation. Supernatants containing the virus stocks were stored at -70°C.

### Immunofluorescence (IF) analysis

FRhK4 cells were grown in 8-well Permanox chamber slides (Nunc, Inc.) at 35°C in a CO_2 _incubator for 24-48 h. Cell culture media was aspirated and the cells were fixed with cold acetone for 30 min at -20°C. Fixed cell monolayers were air-dried, blocked with PBS-2% FBS at room temperature, and stained with a mix of anti-HAV neutralizing murine monoclonal antibodies K2-4F2 and K3-2F2 [35] and fluorescein isothiocyanate (FITC)-conjugated goat anti-mouse antibody (KPL, Inc). Stained slides were air-dried, coverslips were mounted with PermaFluor aqueous medium (Shandon Immunon, PA), and fluorescent micrographs were taken with a Zeiss Axioscope microscope at 400× magnification.

### RT-PCR and nucleotide sequence analysis

RNA was extracted from HAV particles using QIAamp viral RNA mini kit (Qiagen). RT-PCR was carried out using the Superscript-II enzyme kit (Invitrogen). The cDNA was synthesized with primers corresponding to HAV nt position 3715-3738 or 1167-1194. PCR fragments were amplified using primers corresponding to HAV nts 2928-2951 and 3715-3738 or 484-507 and 1167-1194. Amplified DNA fragments were gel purified, and sequenced using the ABI Prism BigDye terminator cycle-sequencing ready reaction kit (Applied Biosystems) and with ABI Prism (model 3100) analyzer (Applied Biosystems).

## Competing interests

The authors declare that they have no competing interests.

The findings and conclusions in this article have not been formally disseminated by the Food and Drug Administration and should not be construed to represent any Agency determination or policy.

## Authors' contributions

KK and GGK conceived the study. KK and GGK designed the experiments and drafted the manuscript. KK and SMN performed the experiments.
